# Stress affects navigation strategies in immersive virtual reality

**DOI:** 10.1038/s41598-024-56048-8

**Published:** 2024-03-11

**Authors:** Apurv Varshney, Mitchell E. Munns, Justin Kasowski, Mantong Zhou, Chuanxiuyue He, Scott T. Grafton, Barry Giesbrecht, Mary Hegarty, Michael Beyeler

**Affiliations:** 1 Department of Computer Science, University of California, Santa Barbara, CA USA; 2 Department of Psychological and Brain Sciences, University of California, Santa Barbara, CA USA; 3 Interdepartmental Graduate Program in Dynamical Neuroscience, University of California, Santa Barbara, CA USA

**Keywords:** Stress and resilience, Human behaviour

## Abstract

There are known individual differences in both the ability to learn the layout of novel environments and the flexibility of strategies for navigating known environments. However, it is unclear how navigational abilities are impacted by high-stress scenarios. Here we used immersive virtual reality (VR) to develop a novel behavioral paradigm to examine navigation under dynamically changing situations. We recruited 48 participants (24 female; ages 17–32) to navigate a virtual maze (7.5 m × 7.5 m). Participants learned the maze by moving along a fixed path past the maze’s landmarks (paintings). Subsequently, participants experienced either a non-stress condition, or a high-stress condition tasking them with navigating the maze. In the high-stress condition, their initial path was blocked, the environment was darkened, threatening music was played, fog obstructed more distal views of the environment, and participants were given a time limit of 20 s with a countdown timer displayed at the top of their screen. On trials where the path was blocked, we found self-reported stress levels and distance traveled increased while trial completion rate decreased (as compared to non-stressed control trials). On unblocked stress trials, participants were less likely to take a shortcut and consequently navigated less efficiently compared to control trials. Participants with more trait spatial anxiety reported more stress and navigated less efficiently. Overall, our results suggest that navigational abilities change considerably under high-stress conditions.

## Introduction

Nearly everyone has experienced the effects of stress on navigation, such as trying to find an unfamiliar location when running late. For emergency responders, every second counts, making stress-induced navigation decisions critical. Despite the real-world importance of studying navigation under stress, this topic has been relatively understudied in the lab.

Navigation is an inherently dynamic and multimodal process^1,2^ that involves learning the layout of new environments (cognitive mapping), updating our position and orientation as we move through space (spatial updating), and planning and executing paths through learned environments to reach goal locations (wayfinding). Key strategies in wayfinding include following well-known paths and exploring novel shortcuts, underpinned by different cognitive and neural representations^3,4^. Stress often promotes a shift from cognitively demanding strategies to habit-based ones^5^. In navigation, stress may cause a switch from hippocampal-dependent behaviors (supporting *configural* or *place* knowledge) to cortico-striatal-dependent ones (supporting *route* or *response* knowledge^6,7^; see also^8^ for a review).

However, the effects of stress on navigation strategies are inconsistent across studies. These discrepancies likely arise from variations in stress induction methods, timing of stress induction, and performance metrics. First, past studies employed a variety of stressors, ranging from the Trier Social Stressor (the participant is interviewed by unfriendly confederates^9,10^), to cold pressor (the participant places their feet in a bucket of ice water^11,12^), physical fatigue^13^, threat of electric shock^14,15^, time pressure^16,17^, monetary loss^18^, and virtual fire^19,20^. Second, whereas some studies induced stress during navigation^14,16,18^, others induced stress post-learning but pre-navigation^13^. Timing of the stressor is critical because there are two stress responses, a rapid “fight or flight” response and a slower hormone-based response^12^. Third, studies differ in how they measure navigation performance, with some studies focusing on wayfinding strategy^13,14,16^, while others focused on navigation success and/or efficiency^11,12,18,21^. Since strategies depend on environmental knowledge and shortcuts are inherently more efficient, we seek to examine the effects of stress on both strategy and efficiency.

Most prior research utilized desktop virtual reality (VR) with limited ecological validity^2^, omitting body-based cues from proprioception and any effect of physical exertion^22–26^. Without a physical cost, people may have less incentive to take a shortcut over a longer learned route^27^. In addition, the degree of immersion in a virtual environment may also affect the stress response.

In this study, we use the Dual Solution Paradigm^3^ to examine the effects of stress on navigation in ambulatory immersive VR. Participants learn a route by walking in an immersive virtual environment, then navigate between landmarks in the virtual environment. During these wayfinding trials, stress was sometimes induced by including time pressure, threatening sounds, fog, and blocking of potential paths. Self-reports were collected on each trial to measure differences in experienced stress levels. Furthermore, spatial anxiety and navigation ability, which differ among individuals, may modulate stress effects on navigation^28,29^. We included measures to understand these effects better. We hypothesized that acute stress would cause participants to shift from a configural strategy to a route strategy. That is, we predicted that participants would choose to take the learned route more often (and navigate less efficiently) in stress trials compared to control trials.

## Methods

### Participants

Forty-eight participants (24 female, ages 18–32) were recruited from undergraduate Psychology courses and received either course credit or a $12 gift card for participating.

### Equipment

A wireless HTC VIVE Pro Eye head-mounted display was used for the immersive virtual reality tasks. The experiment was developed and executed using the Unity game engine on a desktop PC with an Intel i7-11700 k CPU and Nvidia RTX-3080ti GPU.

### Navigation task

**Figure 1:**
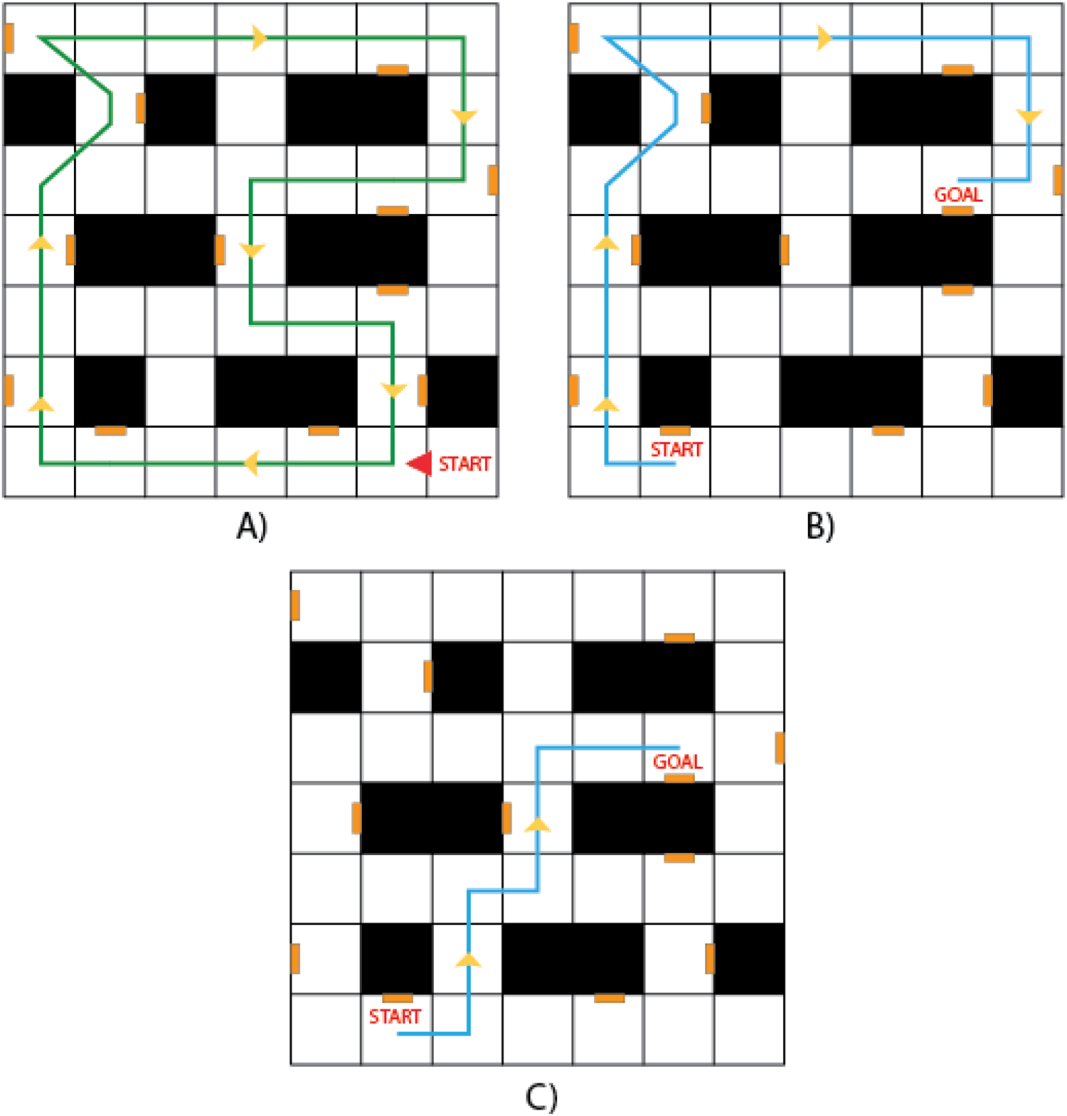
Map of the environment, the landmarks are denoted by the orange boxes, and the black boxes denote the walls. (**A**) The green path indicates the learned route that participants followed during the learning phase. (**B**) and (**C**) Examples of different paths possible for a specific trial with start and goal locations labeled. (**B**) Path based on the learned route, and (**C**) the shortcut path.

A 7.5 m × 7.5 m immersive maze-like environment (see Fig. 1) with 12 landmarks (paintings on the wall), previously used in Dual-Solution Paradigm studies^30^, was adapted for this task. Participants freely walked through the room to navigate while wearing a head-mounted display. It consisted of a learning and wayfinding phase. The learning phase guided participants along a set route (blue path in Fig. 1) that passed by each of the 12 landmarks. Participants said the names of the landmarks aloud the first time on the route, then completed it four more times for a total of five learning trials.

**Figure 2:**
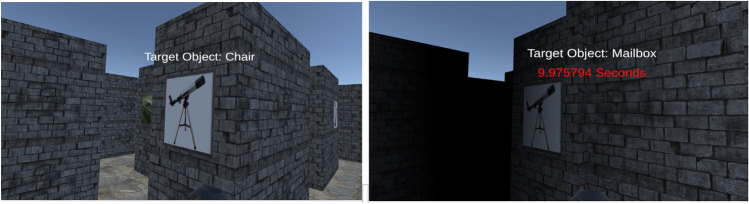
Difference between control (left) and stress (right) trials. For stress trials notice how a wall blocks the path on the right side.

The testing phase had two blocks of wayfinding trials, one block of 12 control trials and one block of 12 stress trials. At the beginning of each trial (both the control and stress) participants first walked to a target (a red pole) in an open environment to disorient them from the maze environment and then were led to another target, at which they could start the trial by pulling the trigger on the controller. They were then placed in the virtual environment at one of the target objects (paintings) and text was shown instructing the participant to go to another target object (see Fig. 2). The control trials had a time limit of 30 s, and ambient white noise playing through the HMD’s headphones (to block out auditory spatial cues from the room). In the stress condition, there were four types of stressors (see Fig. 2): (1) a wall suddenly blocked the path with a loud crashing sound once the participant started walking into the nearest intersection (see Fig. 2), and they were instructed to choose another path; (2) a 20 s time limit with a countdown timer appeared at the top of their visual field with beeping sounds starting when five seconds were left; (3) threatening music played instead of the white noise; (4) the environment was darkened and included fog (obscured vision beyond 2.5 m and the clarity decreased linearly between 1 and 2.5 m) that limited visibility. The wall blocking the path appeared in four to six of the trials in the stress block, while the other stressors were included in all stress trials. These trials will be referred to as “time pressure blocked” and “time pressure non-blocked” for simplicity, although they included multiple stressors.

### Trial-level stress ratings

At the end of each trial, the participants used the VR controller to rate their perceived stress level during the trial on a scale from 1 to 7.

### Pointing task

**Figure 3:**
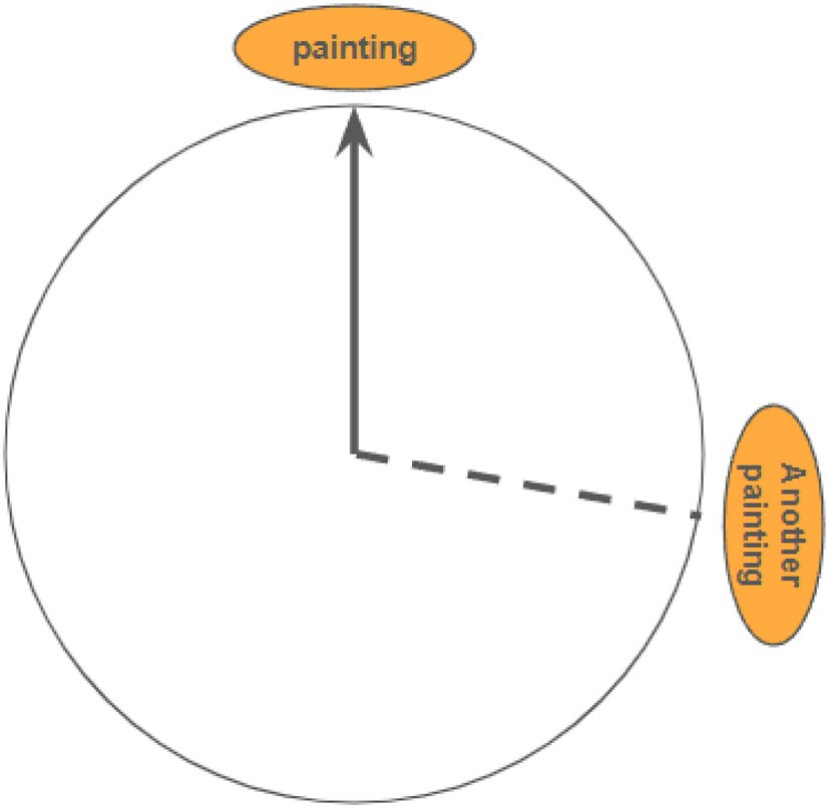
Arrow circle used for the pointing task, participants were instructed to click in the circle to indicate the direction of the target landmark (the dotted line). The participants didn’t see the label of Another painting.

A pointing (i.e., direction estimation) task^30^ was used to measure participants’ (place-based or configural) knowledge of the environment. Participants saw a circle on the screen with one landmark (at the top) and were instructed to imagine they were facing it in the maze and then indicate the direction to a target landmark by clicking in the circle (see Fig. 3). This task had 24 trials that matched the trials of the wayfinding task, using the same starting and target landmark combination for each trial but reversing the starting and target location. The primary performance measure was the absolute angular error of each trial—which could range from 0° to 180°—averaged across trials, with a lower angular error indicating more accurate environmental knowledge.

### Spatial anxiety questionnaire

The Spatial Anxiety Scale^31^ (adapted from Lawton^18^) was employed to measure people’s general spatial anxiety in everyday navigational scenarios. The potential scores range from 1 to 5, and a higher score indicates more anxiety.

### Spatial orientation task

The Spatial Orientation Task (SOT)^32^ measured participants’ perspective-taking and direction-estimation ability. The performance measure is the average absolute angular error with a lower angular error indicating better perspective-taking ability.

### Santa Barbara sense of direction scale

Self-reported sense of direction was measured by a 15-item questionnaire: the Santa Barbara Sense of Direction Scale (SBSOD)^33^. The performance measure is between 1 and 7, where 1 indicates a poor sense of direction and 7 indicates a good sense of direction.

### Procedure

Participants gave informed consent to participate, then were seated at a computer where they completed the spatial orientation task. Following this, they completed a short training on navigating in the immersive virtual environment including time to freely explore a practice virtual environment. The participants next learned the virtual maze by following a guided route indicated by red arrows, which led them to each of the 12 target objects, and were instructed to memorize the locations of these objects. They completed this route five times, then were instructed to retrace the route without any guidance. If they failed to successfully retrace the route, they returned to the starting location and were led by the arrows one more time, followed by another attempt to retrace the route. Then they removed the HMD and completed the pointing task while seated at the computer. After this, they put the HMD back on to perform the wayfinding portion of the navigation task. Participants were randomly assigned to two different groups, one starting with the 12 time pressure trials (*n* = 24) and one starting with the 12 control trials (*n* = 24). Both groups completed all 24 trials. After the wayfinding portion of the navigation task, participants removed the HMD and returned to the computer to complete questionnaires including the Spatial Anxiety questionnaire, and the Santa Barbara Sense of Direction Scale (with the order of these questionnaires counterbalanced across participants).

### Data coding and analysis

There were three measures of wayfinding performance; *Success*, *Excess Distance* and *Shortcut Rate*. *Wayfinding Success was* defined as the percentage of trials in which the participant reached the target object in the time allowed. To compute *Excess Distance* the maze was discretized into 1 m × 1 m nodes, and a pathfinding algorithm was used to determine the number of nodes in the optimal (shortest possible) path for each trial (some trials had multiple optimal paths). Number of nodes in a path was used as the units of distance. *Excess Distance* was then calculated as the ratio of the difference between participant’s walked distance (*W*) and the length of the shortest (optimal) path (*S*) divided by *S* ($Excess \,Distance=\frac{W-S}{S}$)*,* so that a value of 0 would result if the participant always took the most efficient path and larger values indicate less efficient wayfinding. Each trial was coded independently by two coders as either a shortcut (1) or not (0). Interrater reliability was high (mean of Cohen’s Kappa for all trials = 0.93). Shortcut Rate per participant was calculated as number of shortcuts divided by number of successful trials ($Shortcut \,Rate=\frac{\# \,of \,Shortcuts}{Successful \,Trials})$. To avoid confusion, we refer to this measure as Shortcut Rate (although it is commonly called Solution Index in the literature) because the per trial data used in the linear models reflects a binary measure of whether a participant took the shortcut or not. Unsuccessful trials were not included in the shortcut rate or excess distance measures. Optimal paths and shortcut rate were compared only for control and time pressure non-blocked trials, as trials with blocks necessitated taking a longer path (so this would be an unfair comparison). All measures were standardized (mean centered and scaled by 1 standard deviation).

In order to assess how each of these outcome variables and subjective stress ratings were affected by stress, a generalized linear mixed model (GLMM) was fit to the data for each outcome variable, predicted from trial type (control, time pressure non-blocked, or time pressure blocked) and trial order (i.e. the position in the random order in which the trial appeared for a given participant) as fixed effects, and participant number as a random effect. For binary outcome variables (Wayfinding Success and Shortcut per trial), a binomial GLMM (using the function glmer from the R package lme4^34^) was used. For continuous outcome variables (Stress Rating), the function lmer (also from lme4) was used. For Excess Distance (although a continuous variable), the residuals of the model had a non-normal distribution (skewness = 3.15, kurtosis = 19.06), so a robust linear mixed model using the rlmm function from the robustlmm package^35^ was used in order to account for contamination from outliers. In order to calculate a *p-*value for robustlmm, the model generated from the lme4 package was used to obtain Satterthwaite-approximated degrees of freedom, and the *t-*value from the robustlmm model was used^36^.

### Ethical approval

Approved by UCSB’s IRB on 4/28/2023, expires 4/27/2024, IRB Approval Number: 68–23-0173. All research was performed in accordance with the relevant guidelines and regulations of UCSB. Informed consent was obtained from all individual participants included in the study.

## Results

### Stress Rating

**Figure 4:**
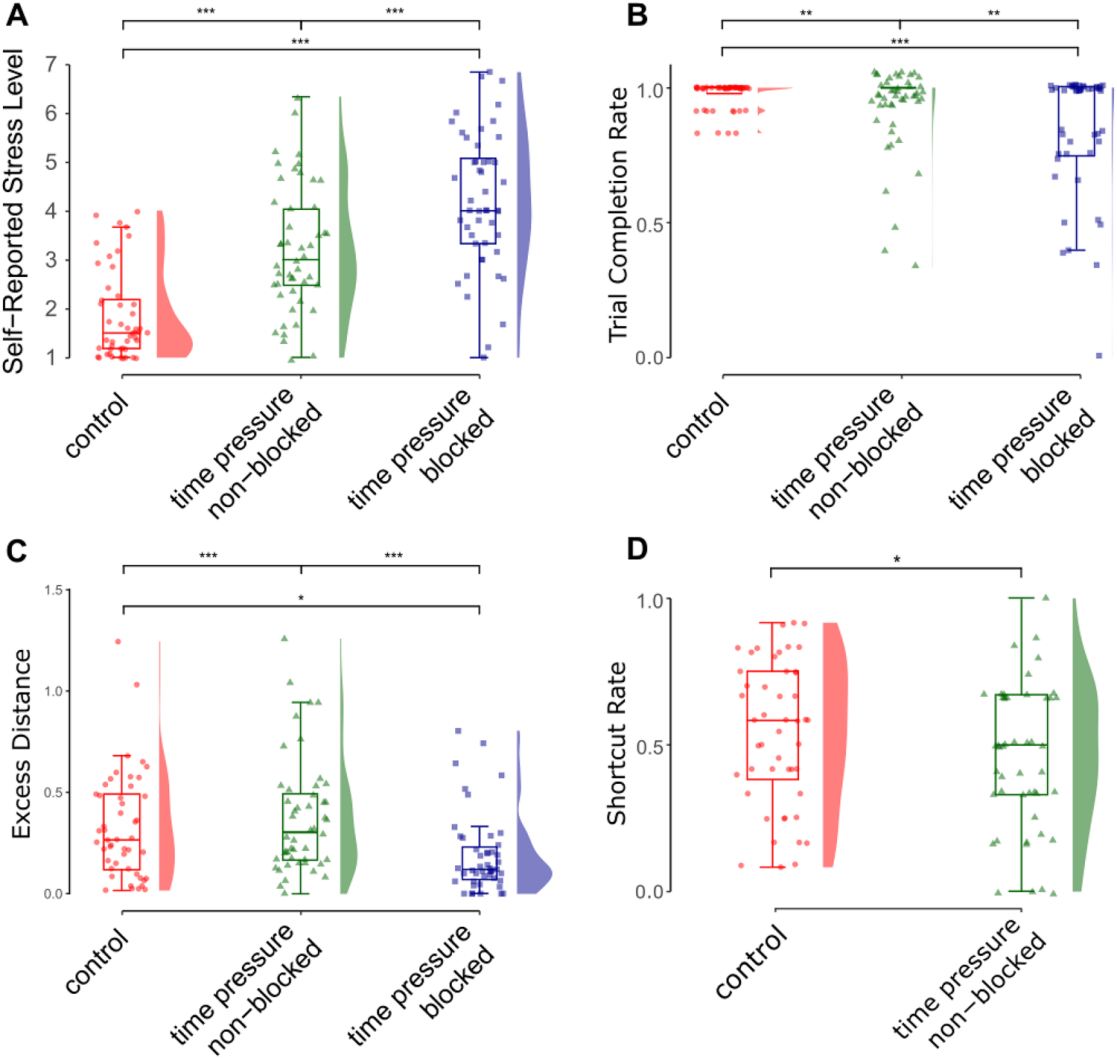
Performance Outcome Plots, Trial Type (control, time pressure non-blocked, time pressure blocked) comparison for: (**A**) Self-reported Stress level, (**B**) Trial Completion Rate, (**C**) Excess Distance, (**D**) Shortcut Rate. Outcomes in the figure were averaged for each participant, but p-values from the linear models above are trial-level.

Participants had higher stress ratings in time pressure non-blocked trials compared to control trials (*b* = 0.811, SE = 0.040, *t* = 20.304, *p* < 0.001), in time pressure blocked trials compared to control trials (*b* = 1.427, SE = 0.042, *t* = 34.153,* p* < 0.001), and in time pressure blocked trials compared to time pressure non-blocked trials (*b* = 0.616, SE = 0.048, *t* = 12.917, *p* < 0.001; see Fig. 4A). This implies that our manipulation was successful in inducing more stress for the two time pressure conditions (blocked and non-blocked). There was a decrease in stress rating as participants advanced through the trials (*b* = − 0.014, SE = 0.002, *t* = − 5.714, *p* < 0.001). Model marginal *R*^2^ = 0.358; conditional *R*^2^ = 0.696.

### Wayfinding success

As shown in Fig. 4B, people were generally successful in finding the goal location in the wayfinding trials (93% success rate overall). Although the stress trials had a lower trial limit (20 s compared to 30 s), only 5.7% of successful control trials took longer than 20 s.Not surprisingly, participants were less successful in time pressure non-blocked trials compared to control trials (*b* = − 0.946, SE = 0.360, *t* = − 2.627, *p* = 0.009), time pressure blocked trials compared to control trials (*b* = − 1.92, SE = 0.332, *t* = − 5.793, *p* < 0.001), and time pressure blocked trials compared to time pressure non-blocked trials (*b* = − 0.976, SE = 0.316, *t* = − 3.094,* p* = 0.002). Success also increased as participants advanced through the trials (*b* = 0.050, SE = 0.021,* t* = 2.399, *p* = 0.016). Model marginal *R*^2^ = 0.040; conditional *R*^2^ = 0.127 (calculated with the R function r.squaredGLMM^37^).

### Excess distance

Participants walked more Excess Distance in time pressure non-blocked trials compared to control trials (*b* = 0.126, SE = 0.038, *t* = 3.326, *p* < 0.001); that is, as predicted, participants traveled less efficient paths when under stress (see Fig. 4C). Participants walked less Excess Distance in time pressure blocked trials compared to control trials (*b* = − 0.095, SE = 0.041, *t* = − 2.323, *p* = 0.02), indicating that they were more efficient after being blocked and forced to change paths. This was interpreted to be due to the reduced number of path choices after a wall block, e.g. if one path is blocked in a four-way intersection, the participant then has a one-in-three chance of choosing the shortest path instead of one-in-four. There was also an effect of trial order such that excess distance decreased, indicating that participants navigated more efficiently as they advanced through the trials (*b* = -0.006, SE = 0.002, *t* = − 2.628, *p* = 0.009). Model marginal *R*^2^ = 0.026; conditional *R*^2^ = 0.170.

### Shortcut rate

Participants took fewer shortcuts in time pressure non-blocked trials compared to control trials (*b* = − 0.384, SE = 0.161, *t* = − 2.379, *p* = 0.017), consistent with our prediction (see Fig. 4D). There was also an increase in shortcuts as participants advanced through the trials (*b* = 0.035, SE = 0.011, *t* = 3.098, *p* = 0.002), consistent with the excess distance measure. Model marginal *R*^2^ = 0.020; conditional *R*^2^ = 0.158.

### Analysis of individual differences

Descriptive statistics for the maze task outcome variables, pointing task performance (absolute pointing error) and self-reported Spatial Anxiety (SA) are shown in Table 1. Success Rate, Excess Distance, and shortcut rate were correlated, although correlations with success rate were relatively low, reflecting less variance in this measure. Interestingly, pointing error was correlated with success rate, excess distance and shortcut rate, suggesting that these measures reflected configural knowledge of the maze, at least in part. Critically, trait spatial anxiety was related to excess distance, taking shortcuts and stress ratings, but not with success rate and pointing. These results suggest that trait anxiety and configural knowledge are independent contributors to wayfinding efficiency. This was confirmed in a linear regression in which Shortcut Rate was uniquely predicted by both pointing error (β = − 0.41, 95% CI[− 0.62, − 0.14]) and spatial anxiety (β = − 0.33, 95% CI[− 0.56, − 0.05]; model adjusted R^2^ = 0.209, *F*(2, 45) = 7.226, *p* = 0.002). Excess distance was also uniquely predicted by both pointing error (β = 0.45, 95% CI[0.20, 0.65]) and spatial anxiety (β = 0.42, 95% CI[0.15, 0.63]; model adjusted R^2^ = 0.308, *F*(2, 45) = 11.48, *p* < 0.001). SBSOD was correlated with spatial anxiety but not with any other outcome measures (and was not a significant predictor in any of the regression models *p* >  = 0.10).

**Table 1:** Means, standard deviations, and correlations with confidence intervals.

Variable	*M(SD)*	Trial completion rate	Excess distance	Shortcut rate	Stress level	Pointing error	Spatial anxiety
Trial completion rate	0.93 (0.09)						
Excess distance	0.32 (0.23)	− 0.37**					
		[− 0.59, − 0.10]					
Shortcut rate	0.51 (0.21)	0.21	− 0.85**				
		[− 0.08, 0.47]	[− 0.91, − 0.74]				
Stress level	2.74 (1.00)	− 0.36*	0.40**	− .29*			
		[− 0.58, − 0.08]	[.14, 0.62]	[− .53, − .01]			
Pointing error	62.08 (21.15)	− 0.44**	0.45**	− .41**	0.33*		
		[− 0.65, − 0.18]	[0.20, 0.65]	[− 0.62, − 0.14]	[0.05, 0.56]		
Spatial anxiety	2.52 (0.55)	− 0.05	0.42**	− 0.33*	0.48**	0.14	
		[− 0.33, 0.24]	[0.15, 0.63]	[− 0.56, − 0.05]	[0.23, 0.67]	[− 0.15, 0.40]	
SBSOD	4.25 (1.02)	0.13	− 0.25	0.24	− 0.20	− 0.08	− 0.52**
		[− 0.16, 0.40]	[− 0.50, 0.03]	[− 0.05, 0.49]	[− 0.46, 0.09]	[− 0.35, 0.21]	[− 0.70, − 0.28]

*M* and *SD* are used to represent mean and standard deviation, respectively. Values in square brackets indicate the 95% confidence interval for each correlation.* indicates *p* < .05. ** indicates *p* < .01.

### Differences in shortcut rate

**Figure 5:**
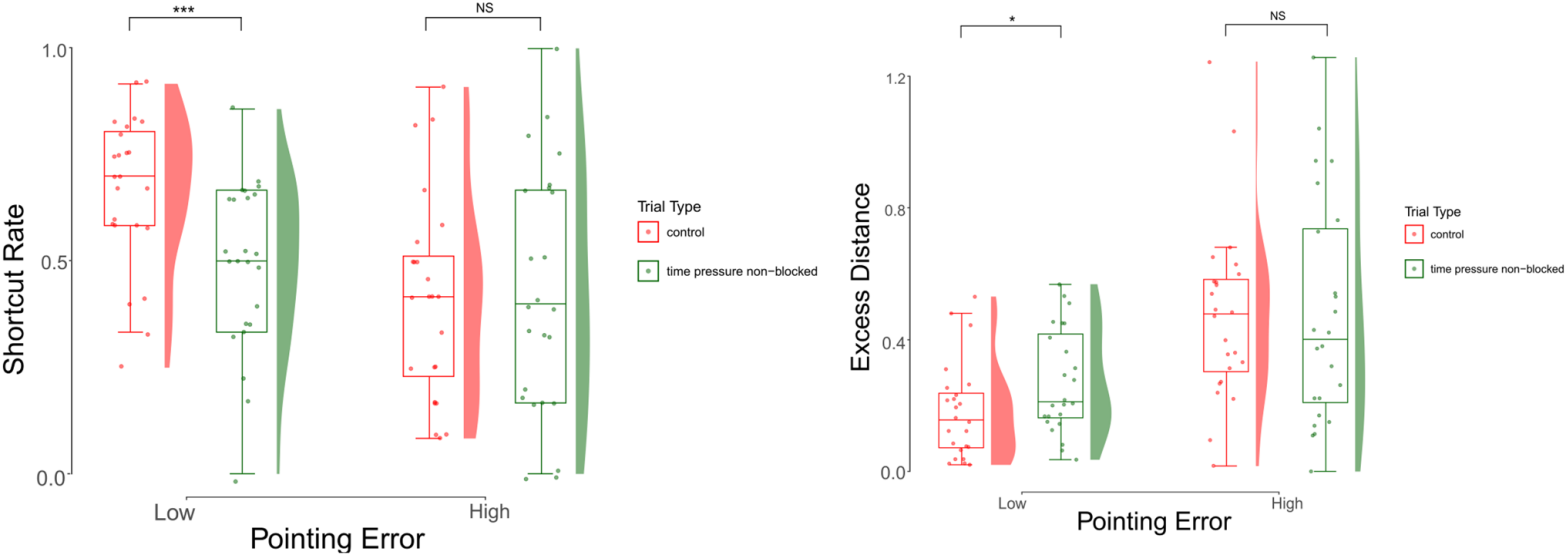
Change in wayfinding performance between trial types for participants with low and high Pointing Error. (**A**) Wayfinding performance measured by Shortcut Rate (higher means more shortcuts taken). (**B**) Wayfinding performance measured by Excess Distance (lower means more efficient navigation).

If stress causes a shift from a cognitive map based strategy to a learned route strategy, we would expect a greater decrease in shortcut rate for those with more developed cognitive maps (lower pointing error). The difference in shortcut rate between control and stressful trials was calculated by subtracting shortcut rate in time pressure non-blocked trials from shortcut rate in control trials (a positive difference means that the participant was more affected by stress). This measure was negatively correlated with pointing error (*r* = − 0.43, *p* = 0.002, 95% CI [− 0.64, − 0.17]) indicating that participants with more developed configural knowledge were more likely to shift from a cognitive map strategy to a route based strategy when under stress. Similarly the difference in excess distance on stress and control trials, (here, a negative difference means that the participant was more affected by stress) was positively correlated with pointing error (*r* = 0.35, *p* = 0.014, 95% CI [0.08, 0.58]). Participants were classified as high or low configural knowledge based on a median split of pointing error. Participants with low pointing error took fewer shortcuts in time pressure non-blocked trials than in control trials (*t*(23) = 3.805, *p* < 0.001, *d* = 0.78) while participants with high pointing error did not differ in navigation strategy between control and time pressure non-blocked trials (*t*(23) = − 0.233, *p* = 0.818, *d* = − 0.05), as shown in Fig. 5A. Similarly, Excess Distance (Fig. 5B) was lower in control trials than time pressure non-blocked trials for participants with high configural knowledge (*t*(23) = − 2.336, *p* = 0.029, *d* = − 0.48) and not different for those with low configural knowledge (*t*(23) = -0.011, *p* = 0.992, *d* = 0.00). These results indicate that stress affected the navigation strategies of participants with high configural knowledge but had no effect on strategies of those with low configural knowledge.

## Discussion

In this study, we used immersive virtual reality (IVR) to examine how stress affects navigation strategy and efficiency. Our findings indicate that under stress, participants navigate less efficiently (Fig. 4C) and favor a learned-route strategy over shortcuts (Fig. 4D)—even in non-blocked stress trials offering the same navigation paths as control trials. Unlike prior desktop virtual environment studies, our IVR approach realistically incorporates the physical cost of longer routes and utilizes proprioceptive cues to enhance spatial learning. Furthermore, our unique stressor (blocked paths) mirrors real-world navigation challenges, such as firefighters facing blocked corridors, making our study more ecologically valid than previous efforts where stressors were not directly related to the task.

Our results support the hypothesis that acute stress leads to a shift in navigation strategy away from flexible, cognitive-based strategies toward habit-based action, which is consistent with earlier findings^14,16^. Our findings may also clarify why other studies^13,21^ did not find such a shift in strategy. First, the timing of the stressor is important to consider. Boone et al.^13^ implemented the stressors (Trier, Cold Pressor and Physical Fatigue) before the navigation task, while the stressors in the studies by Brunye et al.^16^ and Brown et al.^14^ (time pressure and threat of shock) were present during the navigation task itself. One speculative explanation of these results is that the timing of the stressor changes which stress system(s)—either the Hypothalamic–Pituitary–Adrenal (HPA), the Sympathetic-Adrenal-Medulla (SAM) axis or both—is active during the navigation task^38^. Due to the link between cognitive and affective networks in cortex and the HPA, the effects of stress on the HPA can be mediated by cognition^39^. However, stress reactions that activate the SAM (i.e., reactions on a short time scale) may be more reliable in inducing the shift away from hippocampal-dependent strategies toward cortico-striatal-dependent strategies. A possible mechanism for this is the effect of catecholamines (released rapidly during stress) on the basolateral amygdala, leading to a shift in the hippocampus towards memory encoding and away from retrieval^40,41^. It will be important in future research to include physiological measures (including blood pressure, heart rate contractility and cortisol) to better understand the mechanisms underlying shift in navigation strategy due to stress.

Although we found evidence for the shift from shortcut to learned-route strategies, there seems to be a baseline level of learning that has to occur to see a shift due to stress. This was supported by the correlation between change in Shortcut Rate from control to stress and pointing error (*r* = − 0.39). Participants with smaller angular error in the pointing task (indicating a more accurate cognitive map of the environment) took fewer shortcuts in the stress trials compared to control trials (and thus showed a larger shift from flexible cognitive map based strategies to falling back on the learned route). Participants with larger pointing error used the learned-route equally in both conditions, showing no shift in strategy (Fig. 4A) or efficiency (Fig. 4B). This supports the theory that stress inhibits access to configural knowledge and mirrors the finding by Dundon et al.^42^, where, in a task requiring action selection and execution, the more skillful participants (executing) were more likely to revert to a simpler (heuristic) decision strategy.

Trait anxiety was related to state anxiety, indicating that people who are generally more anxious about getting lost or navigating also experience the effects of a stressful navigation scenario more strongly. Interestingly, trait anxiety was also related to excess distance and strategy choice (choosing the learned-route over shortcuts), but was not related to configural knowledge (as measured by the pointing task) or navigation success. These results suggest that individuals with more spatial anxiety are able to construct cognitive maps, but differ in navigation strategies, possibly because they are less confident in their configural knowledge or more risk-averse, so that they are unwilling to take a shortcut when they are uncertain that it will lead to their goal location^27,43^.

In all regression models, a significant effect of trial order was found, indicating that as participants progressed through the wayfinding trials, they either learned the layout better or habituated to the stressors, so that stress effects were reduced. This result is promising because it implies that it may be possible to train people to become resilient to the effects of stress while navigating. Future work could address this by teasing apart which factors lead to the reduction in stress effects, that is, whether it is an effect of learning the environment better through increased exposure, or habituating to the stressors.

### Limitations and future directions

Our self-reported stress measure confirmed the manipulation's effectiveness (Fig. 4A) and correlated as expected with trait anxiety, excess distance, and shortcut rate (Table 1). However, an objective measure of stress may be more sensitive to these effects. To address this limitation, we aim to include a physiological measure of stress in future work.

Using fog as a stressor, which limits visual information (see Fig. 2), might have affected performance in stress trials. Upcoming studies will discern the individual or collective impact of each stressor.

Another limitation is that stressors were applied only post-learning, during testing. However, as highlighted by Schwabe and Wolfe^5^, stressor timing can impact outcomes. Future work should explore the benefits of learning under stress, thereby mirroring testing conditions.

Lastly, route learning only occurred over five repetitions of the route, which may not have been enough to make the route strategy a habit. We did not attempt to have participants overlearn the maze (many repetitions), which in a small environment would likely have resulted in a ceiling effect on navigation performance.

### Conclusion

Consistent with evidence suggesting acute stress shifts from cognitively demanding strategies to habit-based strategies, our study showed that stress worsens navigation performance and causes a preference for learned routes over novel shortcuts, especially for more skillful participants. This mirrors results from Brunye et al.^16^, Brown et al.^14^, and Dundon et al.^42^, but importantly utilizes environment-specific stressors in immersive virtual reality. These findings have implications for real-world navigation scenarios in which stress is an unavoidable component, and can inform future work on how to better learn and navigate an environment under such stress.

## Data Availability

Data and analysis script will be available on GitHub upon acceptance for publication.
